# Treatment of mpox with tecovirimat under expanded access use in the Central African Republic

**DOI:** 10.1016/j.ijid.2026.109027

**Published:** 2026-10

**Authors:** Josephine Bourner, Festus Mbrenga, Elise Pesonel, Aboubacar Soumah, Sandra Garba-Ouangole, Ella Farra, Cyprien Lemon, Christian Malaka, Jake Dunning, Amanda Rojek, Yap Boum, Peter Horby, Emmanuel Nakouné, Piero Olliaro

**Affiliations:** 1 ISARIC, Pandemic Sciences Institute, University of Oxford, Oxford, UK; 2 Institut Pasteur de Bangui, Bangui, Central African Republic

**Keywords:** Mpox, Expanded access, Tecovirimat

## Abstract

**Objectives:**

This programme primarily aimed to provide access to tecovirimat for patients with suspected mpox. Secondary objectives were to describe the clinical and virological features of Clade Ia mpox and assess the safety of tecovirimat. The programme also supported research capacity strengthening, improved case detection through surveillance engagement, and standardised case management.

**Methods:**

An expanded access programme was implemented at Mbaiki District Hospital and Bangui General Hospital. Consenting patients with suspected mpox received oral tecovirimat for 14 days under a standardised protocol. Follow-up visits occurred at day 21 (if PCR-positive at day 14) and day 28 or later. Blood and lesion samples were collected at each visit.

**Results:**

Thirty-one patients were enrolled; 26 (84%) were PCR-positive and 24 (77%) had Clade Ia mpox. Symptoms were heterogeneous, most commonly lymphadenopathy, headache, myalgia, and active lesions. Most symptoms improved by day 14 and the final visit, though lymphadenopathy often persisted. PCR positivity declined over time but remained detectable in some cases. Six serious adverse events occurred in five patients.

**Conclusions:**

While no clear treatment benefit was demonstrated, the programme improved understanding of disease and strengthened clinical and research capacity.

## Highlights

• Clade Ia mpox presentation is heterogeneous.
• Most common symptoms include lymphadenopathy, myalgia, headache and lesions.
• Most symptoms improve by end of treatment; cause unclear (drug or self-limiting).
• MPXV DNA declines over time but may remain detectable beyond day 28.
• Better definition of meaningful outcomes is needed for future trials.

## Introduction

Mpox is a viral zoonotic disease that was historically confined to Central and West Africa – caused respectively by Clade I and Clade II monkeypox virus (MPXV) – with human cases mostly the consequence of spillover events and short chains of human-to-human transmission [1]. The situation has however evolved in the recent past, with the appearance of MPXV subclades IIb and Ib characterised by human-to-human transmission and broader geographical spread, causing the World Health organisation (WHO) to declare two successive public health emergencies of international concern (PHEICs) [2].

While these events attracted substantial public health attention, treatment of mpox remains challenging, with supportive care offering the main care pathway [3,4]. Although tecovirimat received emergency regulatory licencing for the treatment of mpox by the European Medicine Agency [5], it failed to show any advantage over placebo in subsequent randomised controlled trials against Clade Ia [6] and Clade IIb [7]. Brincidofovir and cidofovir have also both been used to treat mpox in small numbers of patients [8] and have been recommended without regulatory authorisation in some jurisdictions [9,10]. Tecovirimat and brincidofovir are also approved by the US FDA under the ‘Animal rule’ for smallpox [11,12]. These drugs however are not widely available in mpox-endemic regions of Central and West Africa, meaning supportive care alone is the only available treatment option outside clinical research programmes.

Mpox Clade Ia is endemic in the Central African Republic (CAR) with a reservoir in small mammals [13]. The country has limited opportunities and capacity to undertake clinical research, due to the remote locations in which the disease is typically detected [14].

To provide access to tecovirimat to patients with mpox (which would otherwise have been unavailable), we conducted an Expanded Access Programme (EAP) with coverage in three prefectures of the Central African Republic (CAR) between 2021 and 2023, before RCTs had been completed elsewhere and the results published. In certain jurisdictions, like in CAR, EAPs act as a regulatory framework within which patients who have a serious illness can access treatment with an Investigational Medicinal Product (IMP) where no appropriate treatment option exists [15].

While the primary objective of the programme was to provide patients with access to treatment with tecovirimat, secondary objectives were to describe the clinical and virological features of patients with mpox who received tecovirimat and describe the safety observations for treated patients. Although not a specific objective under the research protocol, the programme also supported research capacity strengthening to prepare for future studies, increasing early detection of mpox through engagement with the local surveillance system and standardising case management.

## Methods

The methods and results of this EAP are reported according to the CONSORT checklist, which has been adapted for a non-randomised research programme.

The EAP was Sponsored by the University of Oxford and received ethical approval from the Oxford Tropical Research Ethics Committee (OxTREC) (ref: 1-20) and the Comité Ethique et Scientifique de la Faculté des Sciences de la Santé de l’Université de Bangui (ref: 10/UB/FACSS/IPB/CES/023).

The study is registered on ISRCTN (ISRCTN43307947). The protocol is available in Bourner et al., 2024. [16]

A partnership agreement between the University of Oxford, Institut Pasteur de Bangui and Ministry of Health in CAR supported the operationalisation of the programme.

### Design

The EAP provided patients with clinically suspected mpox with access to tecovirimat under a standardised treatment protocol. Treatment was provided to all eligible patients at two health centres, Mbaiki district hospital and Bangui general hospital.

### Participants

Patients with clinically suspected mpox were identified through the national mpox surveillance system and active case finding in the Bangui, Lobaye, Sangha Mbaéré and Manbéré-Kadéï prefectures. Suspected cases were notified to the EAP Principal Investigator (PI) in CAR, who was deployed to the patient’s village or health centre to evaluate the patient for eligibility and invite the patient to join the EAP. Potential participants were provided with relevant study information, given the opportunity to discuss participation, and written informed consent was provided before they were eligible to participate. Blood and lesion samples were collected from all potential participants clinically suspected of mpox infection and tested for MPXV and varicella zoster virus (VZV), according Yto national guidelines (Supplementary File, S1 Text).

Patients who were eligible to join the EAP were those who weighed more than 13 kg, were suspected of having mpox and awaiting laboratory confirmation or had laboratory confirmation by PCR of mpox.

Patients were excluded if they were taking repaglinide or midazolam, or if they had an intolerance to galactose, lactase deficiency or malabsorption of glucose-galactose. Patients who were enrolled on the basis of a clinical suspicion of mpox prior to laboratory confirmation were removed from the study immediately upon receipt of a negative PCR result for MPXV infection.

All participants – regardless of disease severity – were transferred to Mbaiki district hospital or Bangui general hospital for treatment with tecovirimat, routine supportive clinical care, and observation until they were well enough to be discharged.

### Interventions

All patients who consented to participate in the EAP received tecovirimat (TPOXX, SIGA Inc. [17]) in the form of 200 mg immediate-release capsules for 14 days according to the dosing schedule described in Supplementary File Table S1. Tecovirimat was administered by the PI who provided the patient with a meal containing moderate or high fat content 30 minutes before each treatment dose.

After 14 days patients were assessed for discharge. If patients remained clinically unwell or returned a positive PCR result for mpox on Day (D) 14, they would remain in hospital until well enough for discharge and they received a negative PCR result for MPXV DNA.

A blood sample was collected on D1, D4, D8, D14, D21 (for patients who tested positive for mpox at D14 only) and D28 (or a final later timepoint dependent on logistics and is henceforth referred to as the final visit) for all participants. Two lesion swabs and a throat swab sample were collected at the same timepoints for the latter 17 participants enrolled in the study due to an update in the sampling protocol. Pus from the most active lesion and crust samples – when all active lesions had resolved – were collected from the same lesion at each timepoint. All samples were tested by PCR, using the G2RG (generic) assay and C3L (clade-I specific) assay, for MPXV DNA at each timepoint and the overall results with the Ct value were recorded in the CRF.

Clinical data, including signs and symptoms, vital signs and adverse events, were collected daily during hospitalisation and at the final visit.

Voluntary malaria, HIV and pregnancy testing were offered to patients, as appropriate, following consent.

### Outcomes

Clinical outcomes included time to lesion resolution from treatment initiation to day 14, defined as the first day on which all lesions are resorbed, crusted or desquamated and mucosal ulcers have healed, in the absence of any serious complication. Overall clinical status was also assessed from treatment initiation to day 14 and the final visit using an ordinal scale, including a) absence of active lesions and no serious complications, b) active lesions and absence of serious complications, c) serious complications of mpox, d) death.

Virological outcomes were evaluated by the presence of MPXV DNA in lesion, blood, and throat samples at days 4, 8, and 14, as well as at the final visit.

Safety outcomes included the number and type of serious adverse events reported from the time of consent until the final study visit.

Pregnant women were followed-up until the outcome of their pregnancy was known, which was subsequent captured on the CRF.

### Sample size

As this was an EAP with the primary aim of providing treatment to patients, no formal sample size was calculated. However, ethical approval was obtained for the inclusion of up to 100 patients.

### Analysis

The primary analysis population includes only patients who received a positive PCR result for mpox at baseline and received treatment with tecovirimat. There is no comparator group. Prevalence of clinical signs and symptoms are reported as frequency and percentage at relevant timepoints.

Time to lesion resolution is presented as the median number of days for all lesions to have resorbed, crusted or desquamated.

Clinical outcomes are presented as the number and percentage of patients in each category of the ordinal scale described above. Time to lesion resolution was assessed using Kaplan-Meier analysis. The event was defined as documented lesion resolution, with time measured from study entry to resolution or last follow-up. Participants without documented resolution at the end of follow-up were censored. Kaplan–Meier curves with 95% confidence intervals were generated.

Virological outcomes have been summarised as the number and percentage of patients with a positive MPXV PCR result on any sample at D4, D14, D21, and the final visit. The number and percentage of patients with a positive MPXV PCR result at D4, D14, D21 and the final visit has also been summarised with the median Ct value (and standard deviation and range) per sample type (blood, lesion, throat). Cycle threshold (Ct) values were summarised for positive samples and compared across specimen types to evaluate differences in viral load. The association between study day and Ct values was also assessed. Agreement between the G2RG assay and an alternative PCR target (C3L primers) was assessed using the intraclass correlation coefficient (ICC) with 95% confidence intervals.

Serious Adverse Events (SAEs) are reported according to the Medical Dictionary for Regulatory Activities (MedDRA) [18] Preferred Term (PT) and System Organ Class (SOC) and summarised as the number and percentage of patients for whom each event was reported. Causality between the event and tecovirimat was performed independently by the PI and a Medical Monitor (an independent clinician responsible for overseeing study safety). In the event the causality assessment differed between parties, the assessment indicating the stronger relationship to tecovirimat was used for analysis. Causality was categorised as: unrelated to tecovirimat; unlikely to be related; possibly related; probably related; definitely related. Events considered as either unrelated or unlikely to be related to tecovirimat received an overall classification of “unrelated” and events considered as possible, probably or definitely related to tecovirimat received an overall classification of “related.”

Descriptive analyses were performed in R version 4.2.1 [19]. Figure plots were generated using the “ggplot” package.

## Results

The (EAP) was conducted between December 2021 and December 2023 and enrolled 31 patients with suspected mpox. Of these, 26 (84%) had a positive PCR result for mpox and 24 (77%) tested positive for clade I MPXV infection using clade Ia-specific PCR assays. Two patients tested positive for MPXV infection using the generic PCR assays and negative using the clade Ia-specific assay. One participant who tested positive for MPXV also tested positive for VZV. Five patients (16%) tested negative for mpox on baseline samples and were subsequently withdrawn from the study, with treatment discontinued upon receipt of laboratory results. These patients also tested negative for active varicella–zoster virus (VZV) infection by PCR. All 26 participants with PCR-confirmed mpox completed treatment according to the study protocol and were included in the final analysis.

The primary analysis population was 26 participants who tested positive for MPXV, and consisted of 11 male and 15 female participants with a median age of 23 years (Table 1, Supplementary File Table S2). One female participant was pregnant at enrolment. Baseline malaria testing was performed in 23 participants, of whom 21 (91%) tested positive. The median time from symptom onset to treatment initiation was 11 days (range 3-43 days). One participant (4%) tested positive for HIV on D10 of hospitalisation following a period of fatigue, dizziness, headaches, and palpitations, prompting laboratory investigation. The patient started antiretroviral therapy consisting of Dolutegravir-Lamivudine-Tenofovir according to the national treatment protocol.

**Table 1:** Demographic characteristics of the 26 MPXV positive participants enrolled in the study.

*Demographic characteristics*	
Male: female	11:15
Age (years): median (range)	23 (4-42)
Pregnant women: n/N (%) patients tested	1/15 (7%)
***Comorbidities***	
Malaria: n/N (%) patients tested	21/23 (91%)
VZV co-infection: n/N (%) patients tested	1/26 (4%)
HIV infection: n/N (%) patients tested	1/26 (4%)
***General characteristics***	
Time (days) from symptom onset to treatment start: median (range)	11 (3-43)

### Clinical signs and symptoms

Clinical signs and symptoms at baseline were heterogeneous (Table 2). The most commonly reported signs and symptoms were lymphadenopathy, which was present in all participants, headache (25/26, 96% participants), muscle pain (25/26, 96% participants), active lesions (24/26, 92% participants), fever (21/26, 81% participants) and joint pain (21/26, 81% participants).

**Table 2:** Patients’ signs and symptoms at baseline, D14 and the final visit.

*Signs and symptoms*	Baseline	D14	Final visit
	n/N (%) patients	n/N (%) patients	n/N (%) patients
Lymphadenopathy	26 / 26 (100%)	25 / 26 (96%)	14 / 23 (61%)
Headache	25 / 26 (96%)	3 / 26 (12%)	1 / 23 (4%)
Muscle pain	25 / 26 (96%)	4 / 26 (15%)	2 / 23 (9%)
Active lesions	24 / 26 (92%)	6 / 20 (30%)	5 / 19 (26%)
Fever (temperature >38·0°C)	21 / 26 (81%)	1 / 26 (4%)	1 / 23 (4%)
Joint pain	21 / 26 (81%)	3 / 26 (12%)	2 / 23 (9%)
Upper respiratory symptoms	16 / 26 (62%)	3 / 26 (12%)	0 / 23 (0%)
Cough	11 / 26 (42%)	2 / 26 (8%)	0 / 23 (0%)
Lower respiratory symptoms	9 / 26 (35%)	2 / 26 (8%)	0 / 23 (0%)
Eye complications	3 / 12 (25%)	1 / 12 (8%)	0 / 10 (0%)
Keratitis	5 / 26 (19%)	4 / 26 (15%)	4 / 23 (17%)
Pharyngitis	2 / 12 (17%)	0 / 12 (0%)	0 / 10 (0%)
Diarrhoea	3 / 26 (12%)	0 / 26 (0%)	0 / 23 (0%)
Encephalitis	1 / 12 (8%)	1 / 12 (8%)	0 / 10 (0%)
Deep tissue abscess	1 / 12 (8%)	1 / 12 (8%)	1 / 10 (10%)
Vomiting	1 / 26 (4%)	0 / 26 (0%)	0 / 23 (0%)

By the end of treatment (D14) and the final visit, the prevalence of most signs and symptoms had markedly declined, with between zero and four symptoms per patient (median one symptom). Lymphadenopathy was the only symptom that remained common, persisting in 25/26 participants (96%) at D14 and 14/23 participants (61%) at the final visit (Table 2).

Among the 24 participants with active lesions at baseline, the proportion with lesions decreased over time (Table 2; Supplementary File, Table S1). Active lesions were observed in 6/20 participants (30%) at D14 and in 5/19 participants (26%) at the final visit. The median time from treatment initiation to lesion resolution was 8 days (range 1-29 days) (Table 3).

**Table 3:** Outcomes at the final follow-up visit.

Outcome	N (%) patients
Completed the final follow-up visit	23/26 (88%)
PCR positive for MPXV	6/19 (32%)
Persistent active lesions	5/19 (26%)
Time (days) to no active lesions - median (range)	8 (1 - 29)
Continued hospitalisation beyond D14	2 (9%)
Death	1 (4%)

Among 25 lesions analyzed, Kaplan–Meier estimates indicated that 75% had resolved by day 14 (95% CI, 50-87.5%) and 79.2% by day 28 (95% CI, 54.6-90.5%) (Figure 1).

**Figure 1:**
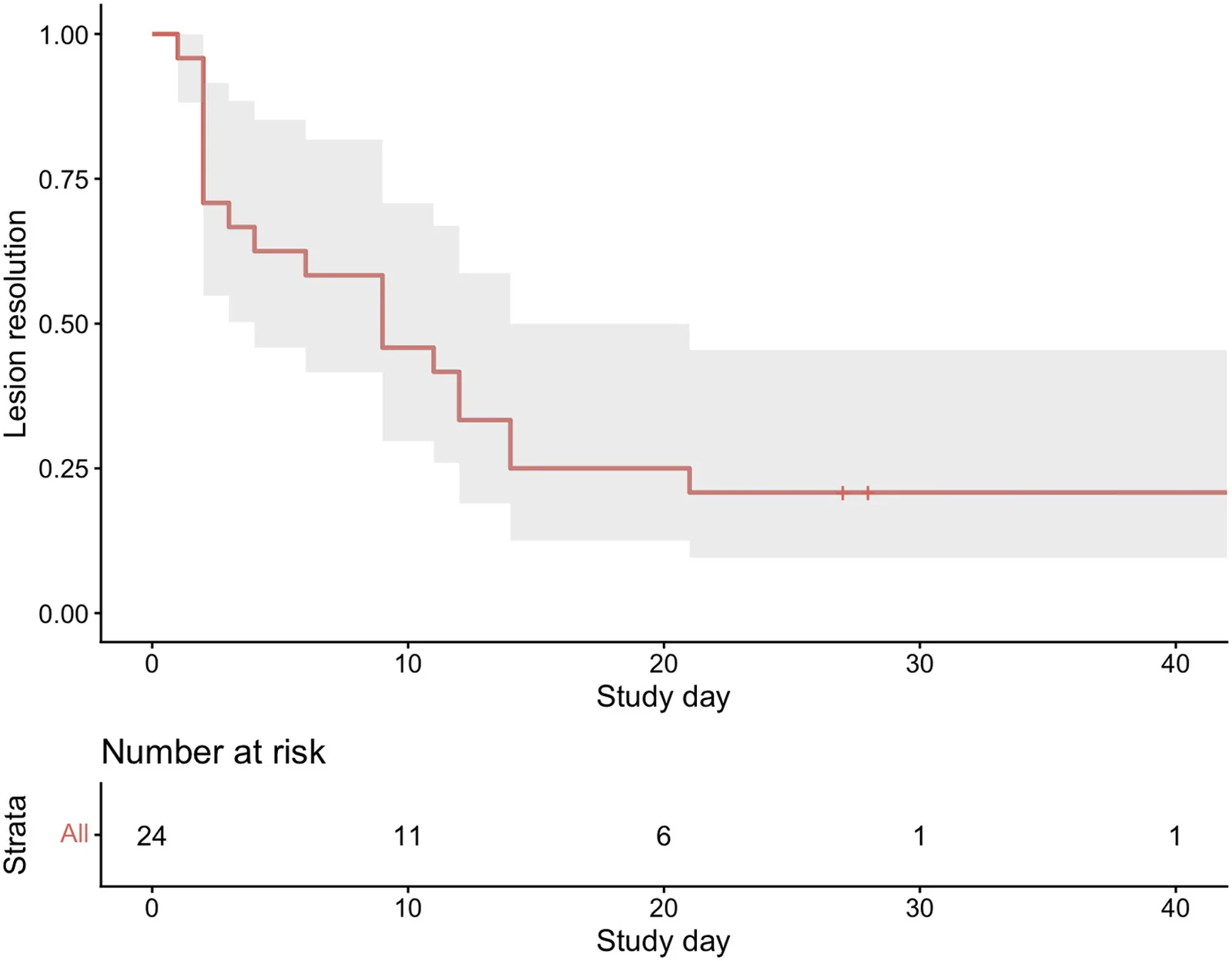
Kaplan–Meier analysis of time to lesion resolution.

Time between symptom onset and treatment initiation was positively correlated with day of lesion resolution (Spearman ρ = 0.69, *p* < 0.001) and day of last PCR-positive test (ρ = 0.61, *p* < 0.001) (Figure 2). Because both variables were measured relative to symptom onset, this association likely reflects the timing of treatment initiation within the disease course rather than a causal effect of treatment timing on outcomes.

**Figure 2:**
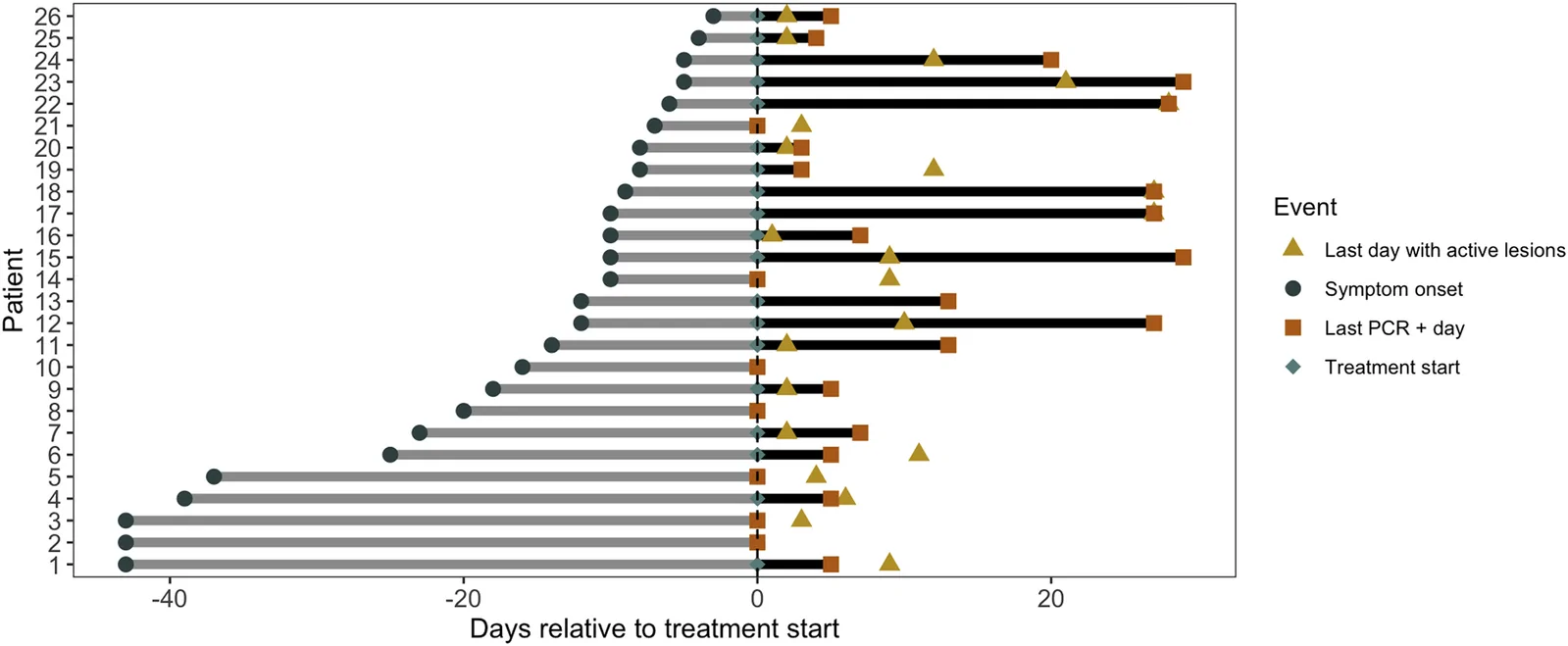
Time between symptom onset, treatment initiation, last PCR+ day and last day with active lesions.

The final follow-up visit was completed by 23/26 participants (88%). Among these, 18 participants (78%) completed a delayed visit beyond D28 due to logistical constraints, with the latest final visit occurring on D50. Two participants (9%) remained hospitalised with serious complications of mpox at D28, and one participant (4%) died on D17, as a result of a homicide. Serious complications observed in two cases included persistent cutaneous and genital lesions (some requiring debridement, suturing, or associated with secondary bacterial infection), persistent cough ascribed to tuberculosis requiring empirical anti-tuberculosis treatment, peritonitis of presumed vaginal origin, acute pneumonia, splenomegaly, and plantar pain.

### Virological results

At baseline, MPXV DNA was detected using the G2RG assay in 20/25 blood samples (80%), 15/17 active lesion samples (88%), and 11/12 scabbed lesion samples (92%) (Table 4; Supplementary File, Table S1). By the end of treatment (D14), 9/19 participants (47%) remained positive on at least one sample type. Positivity was detected in 3/18 blood samples (17%), 6/7 active lesion samples (86%), and 7/9 scabbed lesion samples (78%). At the final visit, 6/19 participants (32%) remained positive on at least one sample type, including 3/19 blood samples (16%), 4/4 active lesion samples (100%), and 5/5 scabbed lesion samples (100%). The Ct values of all PCR-positive crust and lesion samples on D14 and D28 – except one active lesion sample on D28 – were <30. All D14 and D28 Ct values on blood samples were >30 except one on D28.

Among specimens that tested positive by G2RG at baseline, the median cycle threshold (Ct) values were 32.68 for blood samples, 21.76 for active lesion samples, and 21.92 for scabbed lesion samples (Table 4). Ct values differed significantly across sample types. Compared with blood samples, Ct values were lower in both active and scabbed lesion samples, indicating higher viral loads in lesion-derived specimens. Study day was not significantly associated with Ct values.

**Table 4:** G2R-G PCR and CT values (median [min-max]).

	Blood		Crust		Active lesion	
	PCR +	CT-value	PCR +	CT-value	PCR +	CT-value
**D1**	20 / 25 (80%)	32.2 [20.6-41.8]	11 / 12 (92%)	21.4 [15.0-32.0]	15 / 17 (88%)	24.6 [17.2-39.1]
**D14**	3 / 18 (17%)	32.9 [31.4-34.6]	7 / 9 (78%)	18.7 [13.2-26.0]	6 / 7 (86%)	22.8 [15.9-28.2]
**D28**	3 / 19 (16%)	35.0 [28.8-39.9]	5 / 5 (100%)	19.9 [16.2-23.2]	4 / 4 (100%)	22.1 [15.6-31.3]

Agreement between the G2RG assay and results generated using C3L primers was high, with excellent reliability (intraclass correlation coefficient [ICC] 0.925; 95% CI 0.886-0.952).

### Safety

Six SAEs were recorded in five (19%) patients (Supplementary file, Table S3 and S4). Two events – peritonitis and vaginal infection – were observed in one patient. Three events – tuberculosis, vaginal infection and trismus – were present in two patients before enrolment in the EAP and worsened during the course of treatment.

Four events were life-threatening, one event resulted in prolonged hospitalisation and one event was fatal, believed to be the result of homicide (and unrelated to the study). All remaining patients either saw an improvement in their condition or made a full recovery.

All events were determined to be unrelated to tecovirimat.

## Discussion

This EAP provides prospective clinical and virological data on the clinical presentation treatment of PCR-confirmed mpox in the Central African Republic (CAR), as well as on outcomes under the protocolized use of tecovirimat. Compared to the subsequent large clinical trial in the DRC [6], patients in the EAP commenced tecovirimat treatment later after onset of symptoms (PALM 007 mean 6 days; EAP mean 17 days), and approximately one-third of patients in the EAP had persistent general symptoms but no visible active lesion by the time they arrived at the hospital.

Most patients in this EAP cohort treated with tecovirimat showed clinical improvement, a median time to lesion resolution of 8 days, and no serious adverse events attributed to the drug. However, a proportion of patients had persistent PCR positivity or ongoing lesions at the end of follow-up. One death occurred during the course of the programme, which was reportedly unrelated to the disease or treatment.

At baseline, patients presented with a wide range of non-specific symptoms typical of clade I mpox, including universal lymphadenopathy and high frequencies of headache, myalgia and arthralgia. Most patients presented with a characteristic mpox rash (92%). Two patients did not exhibit a rash at enrolment, which may be likely due to the prolonged time between symptom onset and presentation of 37 and 43 days. Other patients with a rash did however have a similarly long delay between symptom onset and presentation. While some of these symptoms may represent a prolonged phase of mpox illness, they may also have been influenced by co-existing conditions, including malaria, which was common in the cohort. As alternative causes of persistent symptoms were not systematically evaluated, attribution of these symptoms to mpox alone is uncertain.

This delay in presenting to a healthcare facility is longer than in many observational cohorts in high-income settings [20,21], reflecting barriers to healthcare access in the Lobaye region of CAR, and may have implications for case management in general, and the assessment of treatment efficacy specifically, in this setting. Whether treatment availability may influence health seeking behaviour requires further assessment.

By day 14 and at the end of follow-up, the prevalence of most clinical signs and symptoms had markedly decreased. However, lesions persisted in a quarter of patients at their last assessment, and two patients remained hospitalised with serious complications. These findings suggest that while most patients experienced favourable clinical trajectories, recovery was prolonged or complicated for several participants. Our findings differ from those of the PALM 007 trial, in which 98% of the cohort had lesion resolution by D28. [6] Persistence of MPXV DNA in lesions and scabs, with or without tecovirimat treatment, is also reported in observational studies of MPXV Clade IIb [20,22], including viral levels compatible with potential for persistent transmissibility – Ct value below 30 used as proxy for culturable virus.

Data collected in this study also demonstrates that viable endpoints for mpox remain unclear [23]. Our data from this cohort, as well as others, confirm that clinical outcomes like “time to lesion resolution”, which has been used in several studies [6,20,21], may not accurately reflect changes in patients’ clinical status over time.

Several challenges with data collection in this study may affect the validity of the results. First, sampling strategies were inconsistent across the cohort. For the first 14 cases, only blood samples were collected; thereafter, attempts were made to collect multiple samples from multiple anatomical sites at each visit. However, the number of oropharyngeal and scabbed lesion samples collected remained low. Second, the final visit, scheduled for D28, occurred much later for 14 participants – up to D50. As a result, the clinical and virological status at D28 is unclear for approximately half of the cohort. Finally, challenging field conditions made it difficult to validate and quality-assure some of the data. These limitations should be considered when interpreting the results. This study shares methodological and inferential challenges of EAPs of Clade IIb mpox conducted in the US [24], as well as similar observational studies [20,21].

In resource-limited settings, programmes such as this may have benefits beyond access to investigational treatment. The EAP supported active identification of potential mpox cases, more structured clinical care through standardised patient assessment, closer clinical follow-up, improved documentation, and systematic monitoring of adverse events. It also enhanced healthcare worker familiarity with mpox and strengthened local capacity to implement research protocols, obtain informed consent, collect high-quality clinical data, and conduct safety monitoring. These capabilities are essential prerequisites for conducting high-quality clinical trials in endemic settings and may leave a lasting legacy beyond the duration of the programme. EAPs are not designed to provide conclusive evidence of efficacy and safety. Here, we aimed at collecting information about the disease presentation and outcomes under treatment through protocolized use of tecovirimat, and providing better care in a setting where minimal care was available. Earlier diagnosis and strengthened clinical infrastructure in endemic regions should be prioritised to support healthcare access and future equitable clinical trial participation. Our study also underscores the need for a clearer understanding of meaningful patient outcomes for future clinical trials to evaluate much-needed effective treatments.

## Funding

SIGA donated 100 treatments for use under the EAP. The EAP was financed with: core funds of Institut Pasteur de Bangui (IPB); financial support to IPB from SIGA; the University of Oxford under the joint UK Foreign, Commonwealth and Development Office and Wellcome grant (215,091/Z/18/Z) and the Bill & Melinda Gates Foundation (OPP1209135), for which PH was the recipient of both grants. The funders and Sponsors had no role in the design, investigation or analysis of the study.

## Ethical approval statement

This study was Sponsored by the University of Oxford and received ethical approval from the Oxford Tropical Research Ethics Committee (OxTREC) (ref: 1-20) and the Comité Ethique et Scientifique de la Faculté des Sciences de la Santé de l’Université de Bangui (ref: 10/UB/FACSS/IPB/CES/023). The study is registered on ISRCTN (ISRCTN43307947).

## CRediT authorship contribution statement

**Josephine Bourner:** Conceptualization, Data curation, Formal analysis, Methodology, Project administration, Writing – original draft, Writing – review & editing. **Festus Mbrenga:** Investigation, Supervision, Writing – review & editing. **Elise Pesonel:** Data curation, Formal analysis, Project administration, Validation, Writing – original draft, Writing – review & editing. **Aboubacar Soumah:** Investigation, Project administration, Writing – review & editing. **Sandra Garba-Ouangole:** Investigation, Writing – review & editing. **Ella Farra:** Investigation, Writing – review & editing. **Cyprien Lemon:** Data curation, Writing – review & editing. **Christian Malaka:** Investigation, Writing – review & editing. **Jake Dunning:** Investigation, Supervision, Writing – review & editing. **Amanda Rojek:** Resources, Supervision, Writing – review & editing. **Yap Boum II:** Resources, Supervision, Writing – review & editing. **Peter Horby:** Resources, Supervision, Writing – review & editing, Conceptualization, Funding acquisition, Methodology. **Emmanuel Nakouné:** Conceptualization, Resources, Supervision, Writing – review & editing. **Piero Olliaro:** Conceptualization, Formal analysis, Methodology, Resources, Supervision, Writing – review & editing.

## Declaration of competing interest

The authors declare the following financial interests/personal relationships which may be considered as potential competing interests: JD received remuneration for attending a single scientific advisory board for Siga Technologies in January 2026. The remaining authors declare no conflicts of interest.

## Appendix Supplementary materials

Image, application 1
