# Supplementary material for: Treatment of mpox with tecovirimat under expanded access use in the Central African Republic
Source: Int J Infect Dis. 2026 Oct;171:None. doi: 10.1016/j.ijid.2026.109027 (PMC13615899; doi:10.1016/j.ijid.2026.109027)
Supplement: Supplementary file 1 [file mmc1.docx]

**Supplementary file**

**Text S1. National guidelines for mpox surveillance and testing in CAR.**

The Ministry of Health and Population set up a passive surveillance program for mpox in 2010. Under this program, specimens are collected from all suspected cases with illness meeting the standardised case definition (below), which is disseminated to all health professionals in CAR through regular training sessions and posters displayed in health facilities. Specimens are sent for biologic confirmation by PCR to the national reference laboratory at Institut Pasteur de Bangui (IPB). Whenever possible, contact tracing is conducted after identification of confirmed cases.

Case Definitions for Suspected and Confirmed Mpox

The following mpox case definitions are communicated to health professionals across the Central African Republic in training sessions. Flyers containing the case definitions are provided to participants during the training for display at their health facilities in locations visible to anyone who enters.

Suspected case:

Any patient who presents to a health facility with the following signs and symptoms:

- Fever or suspected fever
- Cutaneous rash over the body, particularly on the palms of the hands or soles of the feet
- Adenopathy

Confirmed case:

Any patient whose sample is confirmed by PCR for mpox in the laboratory.

Procedure for Mpox Sample Collection

The standard procedures for collection mpox samples are as follows:

- Written or oral consent is obtained from the patient (or a legal guardian if the patient is a minor)
- Completion of the case notification form provided under the national surveillance program
- Trained healthcare personnel collect the following sample(s):
  - Blood is collected at the onset of the acute phase of the disease or during the late phase after all lesions have desquamated
  - If lesions have not scabbed, a lesion swab is collected
  - If lesion have scabbed, the crusts are removed when the lesions have dried but are still stuck to the body
- All samples are sent to the national reference laboratory at Institut Pasteur de Bangui to be tested for VZV and MPXV by PCR using G2R-G and C3L primers.

Each specimen received at IPB is tested for MPXV by real-time PCR. After specimen processing, 200 µL of each sample are extracted by using the QIAamp Viral DNA Mini Kit (QIAGEN, <https://www.qiagen.com>) according to the manufacturer’s instructions. The reactions are performed in 25 µL volume containing 12.5 µL of TaqMan Universal PCR Master Mix (Thermo Fisher Scientific, <https://www.thermofisher.com>), 4.5 µL of nuclease-free water (Thermo Fisher), 1 µL of each 10 µmol/L primer developed by TaqMan technology (Thermo Fisher), using the generic primer (G2RG) and clade I–specific (C3L) primers and 5 µL of extracted DNA. On the basis of these same concentrations, varicella zoster virus (VZV) primers (VZV open reading frame 63) are also used.

**Table S1. Tecovirimat dosing schedule**

| **Weight** | **Dosage** |
| --- | --- |
| **Adults above 40kg** | |
| 40kg to 120kg | 600mg twice daily |
| ≥120kg | 600mg three times daily |
| **Adults below 40kg and children** | |
| 13kg to 24kg | 200mg twice daily |
| 25kg to 39kg | 400mg twice daily |
| ≥40kg | 600mg twice daily |

**Table S2. Summary of participant characteristics at baseline (D1), D14, D21 and the final study visit.**

| **Participant** | **Age**  **(Years)** | **Sex** | **VZV PCR result at baseline** | **N days between symptom onset and enrolment** | **Study day** | **Mpox PCR result (G2RG)** | | | | **Active lesions** |
| --- | --- | --- | --- | --- | --- | --- | --- | --- | --- | --- |
|  |  |  |  |  |  | **Blood**  **[Ct value]** | **Pus**  **[Ct value]** | **Crust**  **[Ct value]** | **Saliva**  **[Ct value]** |  |
| 1 | 27 | F | NEGATIVE | 18 | 1 | +  [37.6] | +  [29.46] | NA | NA | Yes |
|  |  |  |  |  | 14 | - | NA | NA | NA | No |
|  |  |  |  |  | 21 | NA | NA | NA | NA | NA |
|  |  |  |  |  | Final visit | - | NA | NA | NA | No |
| 2 | 22 | M | NEGATIVE | 39 | 1 | +  [34.86] | NA | NA | NA | Yes |
|  |  |  |  |  | 14 | - | NA | NA | NA | No |
|  |  |  |  |  | 21 | NA | NA | NA | NA | NA |
|  |  |  |  |  | Final visit | - | NA | NA | NA | No |
| 3 | 17 | F | NEGATIVE | 25 | 1 | +  [27.41] | NA | NA | NA | Yes |
|  |  |  |  |  | 14 | - | NA | NA | NA | No |
|  |  |  |  |  | 21 | NA | NA | NA | NA | NA |
|  |  |  |  |  | Final visit | - | NA | NA | NA | No |
| 4 | 4 | F | NEGATIVE | 43 | 1 | +  [20.6] | NA | NA | NA | Yes |
|  |  |  |  |  | 14 | - | NA | NA | NA | No |
|  |  |  |  |  | 21 | NA | NA | NA | NA | NA |
|  |  |  |  |  | Final visit | - | NA | NA | NA | No |
| 5 | 28 | F | NEGATIVE | 4 | 1 | +  [33.75] | +  [36.60] | NA | NA | Yes |
|  |  |  |  |  | 14 | - | NA | NA | NA | No |
|  |  |  |  |  | 21 | NA | NA | NA | NA | NA |
|  |  |  |  |  | Final visit | - | NA | NA | NA | No |
| 6 | 23 | M | NEGATIVE | 3 | 1 | +  [31.58] | +  [39.13] | NA | NA | Yes |
|  |  |  |  |  | 14 | - | NA | NA | NA | No |
|  |  |  |  |  | 21 | NA | NA | NA | NA | NA |
|  |  |  |  |  | Final visit | - | NA | NA | NA | No |
| 7 | 38 | F | NEGATIVE | 43 | 1 | +  [29.47] | NA | NA | NA | No |
|  |  |  |  |  | 14 | - | NA | NA | NA | No |
|  |  |  |  |  | 21 | NA | NA | NA | NA | NA |
|  |  |  |  |  | Final visit | - | NA | NA | NA | No |
| 8 | 23 | F | NEGATIVE | 8 | 1 | +  [33.69] | NA | NA | NA | Yes |
|  |  |  |  |  | 14 | - | NA | NA | NA | No |
|  |  |  |  |  | 21 | NA | NA | NA | NA | NA |
|  |  |  |  |  | Final visit | - | NA | NA | NA | No |
| 9 | 5 | F | NEGATIVE | 43 | 1 | +  [26.92] | NA | NA | NA | Yes |
|  |  |  |  |  | 14 | NA | NA | NA | NA | Yes |
|  |  |  |  |  | 21 | NA | NA | NA | NA | NA |
|  |  |  |  |  | Final visit | - | NA | NA | NA | Yes |
| 10 | 25 | F | NEGATIVE | 10 | 1 | +  [40.73] | NA | NA | NA | Yes |
|  |  |  |  |  | 14 | NA | NA | NA | NA | No |
|  |  |  |  |  | 21 | NA | NA | NA | NA | NA |
|  |  |  |  |  | Final visit | - | NA | NA | NA | No |
| 11 | 35 | M | NEGATIVE | 37 | 1 | +  [41.78] | NA | NA | NA | No |
|  |  |  |  |  | 14 | NA | NA | NA | NA | No |
|  |  |  |  |  | 21 | NA | NA | NA | NA | NA |
|  |  |  |  |  | Final visit | - | NA | NA | NA | No |
| 12 | 16 | M | NEGATIVE | 16 | 1 | - | +  [18.55] | NA | NA | Yes |
|  |  |  |  |  | 14 | NA | NA | NA | NA | No |
|  |  |  |  |  | 21 | NA | NA | NA | NA | NA |
|  |  |  |  |  | Final visit | - | NA | NA | NA | No |
| 13 | 20 | F | NEGATIVE | 20 | 1 | - | +  [20.75] | NA | NA | Yes |
|  |  |  |  |  | 14 | NA | NA | NA | NA | No |
|  |  |  |  |  | 21 | NA | NA | NA | NA | NA |
|  |  |  |  |  | Final visit | - | NA | NA | NA | No |
| 14 | 8 | F | NEGATIVE | 5 | 1 | +  [29.28] | NA | NA | NA | Yes |
|  |  |  |  |  | 14 | +  [32.62] | NA | NA | NA | Yes |
|  |  |  |  |  | 21 | +  [39.5] | NA | NA | NA | Yes |
|  |  |  |  |  | Final visit | +  [39.88] | NA | NA | NA | No |
| 15 | 24 | M | NEGATIVE | 12 | 1 | +  [32.11] | +  [17.55] | +  [15.31] | +  [30.15] | Yes |
|  |  |  |  |  | 14 | - | +  [27.62] | +  [20.43] | - | Yes |
|  |  |  |  |  | 21 | - | - | +  [33.36] | NA | NA |
|  |  |  |  |  | Final visit | - | +  [17.61] | +  [21.65] | NA | Yes |
| 16 | 37 | M | NEGATIVE | 10 | 1 | - | +  [18.99] | +  [17.75] | +  [35.93] | Yes |
|  |  |  |  |  | 14 | NA | +  [24.87] | +  [17.81] | NA | No |
|  |  |  |  |  | 21 | NA | +  [26.43] | +  [29.55] | NA | No |
|  |  |  |  |  | Final visit | NA | NA | +  [21.23] | NA | No |
| 17 | 42 | M | POSITIVE | 7 | 1 | NA | - | +  [31.45] | - | Yes |
|  |  |  |  |  | 14 | NA | NA | NA | NA | NA |
|  |  |  |  |  | 21 | NA | NA | NA | NA | NA |
|  |  |  |  |  | Final visit | NA | NA | NA | NA | NA |
| 18 | 23 | F | NEGATIVE | 8 | 1 | +  [35.5] | +  [21.76] | +  [21.93] | - | Yes |
|  |  |  |  |  | 14 | NA | NA | NA | NA | NA |
|  |  |  |  |  | 21 | NA | NA | NA | NA | NA |
|  |  |  |  |  | Final visit | NA | NA | NA | NA | NA |
| 19 | 37 | M | NEGATIVE | 23 | 1 | - | +  [32.68] | +  [30.74] | - | Yes |
|  |  |  |  |  | 14 | - | NA | - | - | NA |
|  |  |  |  |  | 21 | NA | NA | NA | NA | NA |
|  |  |  |  |  | Final visit | NA | NA | NA | NA | NA |
| 20 | 16 | F | NEGATIVE | 10 | 1 | - | +  [22.83] | +  [22.92] | +  [36.53] | Yes |
|  |  |  |  |  | 14 | - | NA | NA | NA | NA |
|  |  |  |  |  | 21 | NA | NA | NA | NA | NA |
|  |  |  |  |  | Final visit | NA | NA | NA | NA | NA |
| 21 | 22 | M | NEGATIVE | 14 | 1 | +  [33.95] | +  [27.83] | +  [25.41] | - | Yes |
|  |  |  |  |  | 14 | - | +  [15.91] | +  [25.92] | - | NA |
|  |  |  |  |  | 21 | NA | NA | NA | NA | NA |
|  |  |  |  |  | Final visit | NA | NA | NA | NA | NA |
| 22 | 13 | F | NEGATIVE | 12 | 1 | +  [33.25] | +  [25.73] | +  [21.01] | - | Yes |
|  |  |  |  |  | 14 | +  [34.63] | - | - | NA | NA |
|  |  |  |  |  | 21 | NA | NA | NA | NA | NA |
|  |  |  |  |  | Final visit | NA | NA | NA | NA | NA |
| 23 | 15 | F | NEGATIVE | 9 | 1 | +  [29.49] | +  [20.02] | +  [21.92] | +  [28.72] | Yes |
|  |  |  |  |  | 14 | - | +  [22.64] | +  [21.72] | - | Yes |
|  |  |  |  |  | 21 | - | +  [17.51] | - | - | Yes |
|  |  |  |  |  | Final visit | +  [28.78] | +  [15.91] | +  [16.81] | - | Yes |
| 24 | 39 | M | NEGATIVE | 6 | 1 | +  [29.77] | +  [17.2] | +  [15.36] | +  [24.93] | Yes |
|  |  |  |  |  | 14 | - | +  [18.53] | +  [16.46] | - | Yes |
|  |  |  |  |  | 21 | - | +  [16.46] | +  [17.44] | - | Yes |
|  |  |  |  |  | Final visit | - | +  [31.31] | +  [27.15] | - | Yes |
| 25 | 23 | M | NEGATIVE | 5 | 1 | +  [35.38] | - | - | +  [31.75] | Yes |
|  |  |  |  |  | 14 | - | NA | +  [17.7] | - | No |
|  |  |  |  |  | 21 | - | +  [32.72] | +  [29.09] | - | No |
|  |  |  |  |  | Final visit | - | NA | NA | - | NA |
| 26 | 38 | F | NEGATIVE | 10 | 1 | +  [29.82] | +  [17.23] | +  [15.98] | - | Yes |
|  |  |  |  |  | 14 | +  [31.43] | +  [17.82] | +  [13.18] | +  [39.96] | Yes |
|  |  |  |  |  | 21 | - | +  [20.17] | +  [18] | +  [35.28] | Yes |
|  |  |  |  |  | Final visit | +  [36.19] | +  [19.44] | +  [16.17] | - | Yes |

**Table S3. Summary of Serious Adverse Events (SAEs) between baseline and D28**

| **MedDRA SOC**  MedDRA Preferred Term | **N (%) patients** | **Severity^1^** | **Outcome** | **Causality to tecovirimat** |
| --- | --- | --- | --- | --- |
| **Blood and lymphatic system disorders** | | | | |
| Anaemia | 1 (4%) | 4 | Resolved | Not related |
| **General disorders and administration site conditions** | | | | |
| Death (unexplained) | 1 (4%) | 5 | Fatal | Not related |
| **Infections and infestations** | | | | |
| Peritonitis | 1 (4%) | 4 | Resolved | Not related |
| Tuberculosis | 1 (4%) | 2 | Improved | Not related |
| Vaginal infection | 1 (4%) | 4 | Resolved | Not related |
| **Musculoskeletal and connective tissue disorders** | | | | |
| Trismus | 1 (4%) | 4 | Resolved | Not related |

^1^ 1 = Mild; 2 = Moderate; 3 = Severe; 4 = Life-threatening; 5 = Fatal

**Table S4. Summary Serious Adverse Events occurring in the study between consent and the final visit.**

| **Event** | **Grade** | **Seriousness** | **Study day on which the event started** | **Study day on which the event ended** | **Outcome** | **Causality with tecovirimat** |
| --- | --- | --- | --- | --- | --- | --- |
| Anaemia | 4 | Life-threatening | D9 | D14 | Recovery without sequelae | Not related |
| Unexplained death^1^ | 5 | Death | D48 | D48 | Death | Unlikely to be related |
| Tuberculosis (recurrent) | 2 | New or prolonged hospitalisation | The event started two years before the start of treatment and worsened on D6 of treatment | D15 | Improvement in condition | Not related |
| Vaginitis ^2^ | 4 | Life-threatening | The event started 12 days before treatment and worsened on D2 of treatment | D17 | Recovery without sequelae | Not related |
| Peritonitis ^2^ | 4 | Life-threatening | D12 | D26 | Recovery without sequelae | Unlikely to be related |
| Trismus | 4 | Life-threatening | The event started three days before the start of treatment and worsened on D4 of treatment | D4 | Recovery without sequelae | Not related |

^1^ Death appears to be result of homicide

^2^ Events reported for the same patient
